# Supplementary material for: Free-Standing ZnO:Mo Nanorods Exposed to Hydrogen or Oxygen Plasma: Influence on the Intrinsic and Extrinsic Defect States
Source: Materials (Basel). 2022 Mar 18;15(6):2261. doi: 10.3390/ma15062261 (PMC8949513; doi:10.3390/ma15062261)
Supplement: Supplementary file 1 [file materials-15-02261-s001.zip › materials-1614572-supplementary.pdf]

materials

MDPI

Supplementary information for the paper entitled: **"Free-standing ZnO:Mo Nanorods Exposed to Hydrogen or Oxygen Plasma: Influence on the Intrinsic and Extrinsic Defect States"**

**Maksym Buryi [1], Zdeněk Remeš [1,*], Vladimir Babin [1], Sergii Chertopalov [1], Kateřina Děcká [1,2], Filip Dominec [1], Júlia Mičová [3] and Neda Neykova [1,4,*]**

[1] Institute of Physics of the Czech Academy of Sciences, Na Slovance 1999/2, 182 00 Prague, Czech Republic; buryi@fzu.cz (M.B.); babinv@fzu.cz (V.B.); chertopalov@fzu.cz (S.C.); decka@fzu.cz (K.D.) dominecf@fzu.cz (F.D.)
[2] Department of Nuclear Chemistry, Faculty of Nuclear Sciences and Physical Engineering, Czech Technical University in Prague, Břehová 7, 115 19 Prague, Czech Republic
[3] Institute of Chemistry SAS, Dúbravská cesta 9, 845 38 Bratislava, Slovakia; chemjumi@savba.sk
[4] Centre for Advanced Photovoltaics, Faculty for Electrical Engineering, Czech Technical University in Prague, Technická 2, 166 27 Prague, Czech Republic
* Correspondence: remes@fzu.cz (Z.R.); neykova@fzu.cz (N.N.) Tel.: +420-220-318-540 (Z.R.); +420-220-318-516 (N.N.)

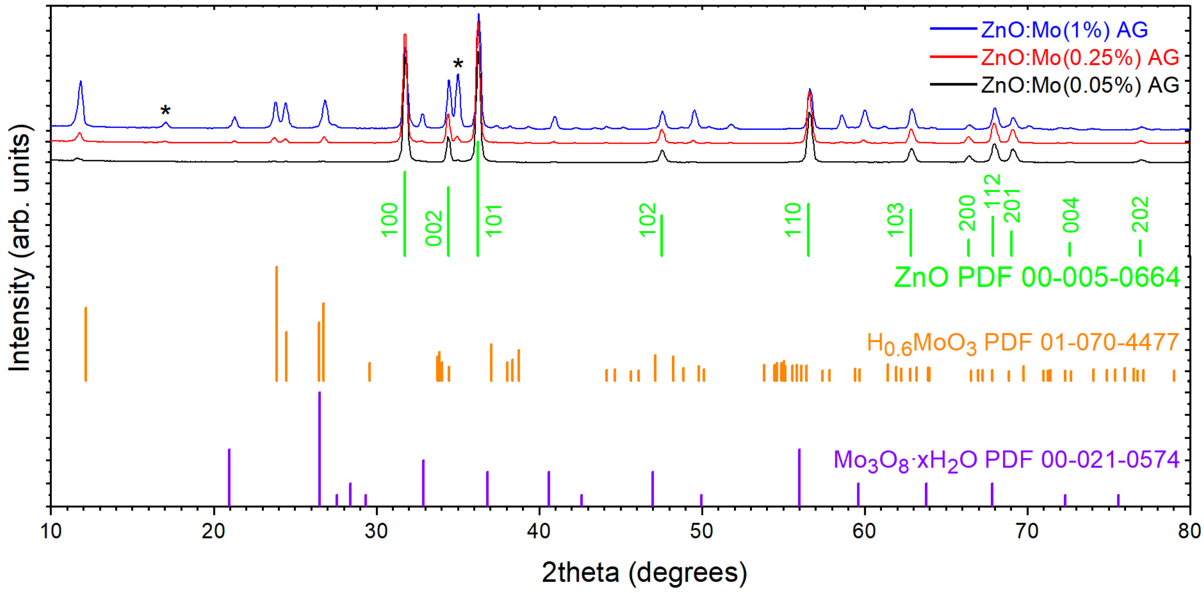


**Figure S1.** XRD pattern of the as grown ZnO:Mo(0.05, 0.25 and 1%). The reflections of the hexagonal Wurtzite ZnO (the peaks are indicated), $H_{0.6}MoO_3$ and $Mo_3O_8{\cdot}xH_2O$ phases are indicated according to the ICDD PDF-2 database. Asterisks indicate reflections which might originate from $Zn_2Mo_3O_8$ oriented along {100}.

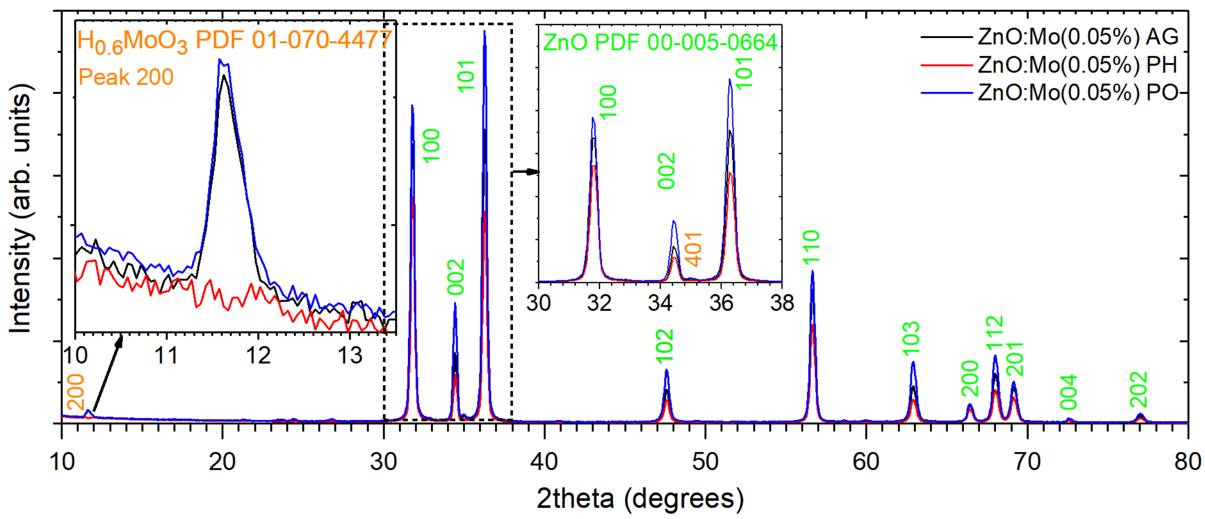


**Figure S2.** XRD pattern of the ZnO:Mo(0.05%) samples, as grown, hydrogen or oxygen plasma treated. The peaks of ZnO phase are stressed with green marks whereas those visible of the $H_{0.6}MoO_3$ phase are stressed by orange marks. The leftmost inset shows the peak 200 of the $H_{0.6}MoO_3$ phase whereas the rightmost inset shows peaks 100, 002 and 101 of the ZnO phase and the peak 401 of the $H_{0.6}MoO_3$ phase. This is in accordance with the ICDD PDF-2 database.

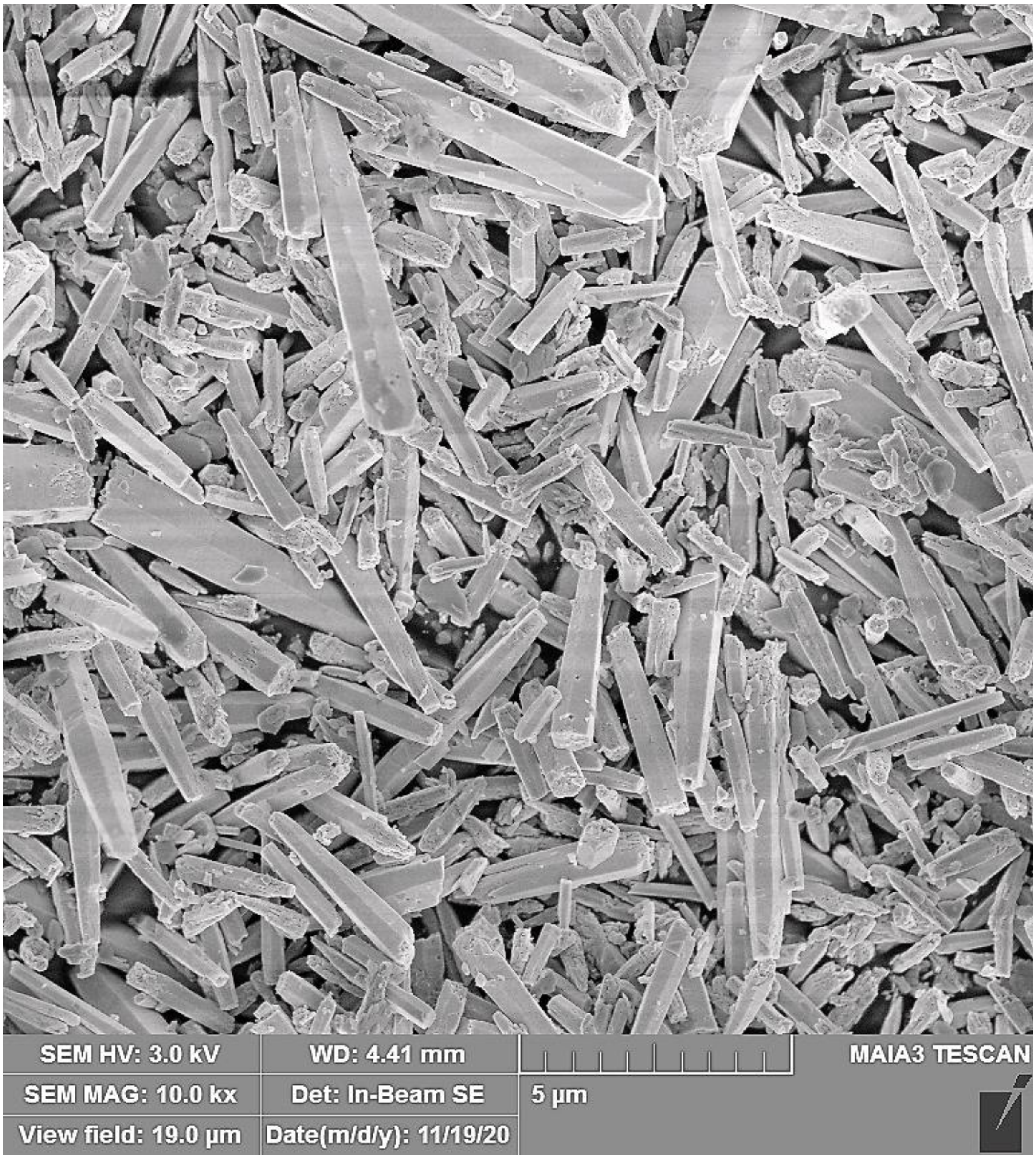


**Figure S3.** Original SEM image of the as grown ZnO:Mo(0.05%) sample.

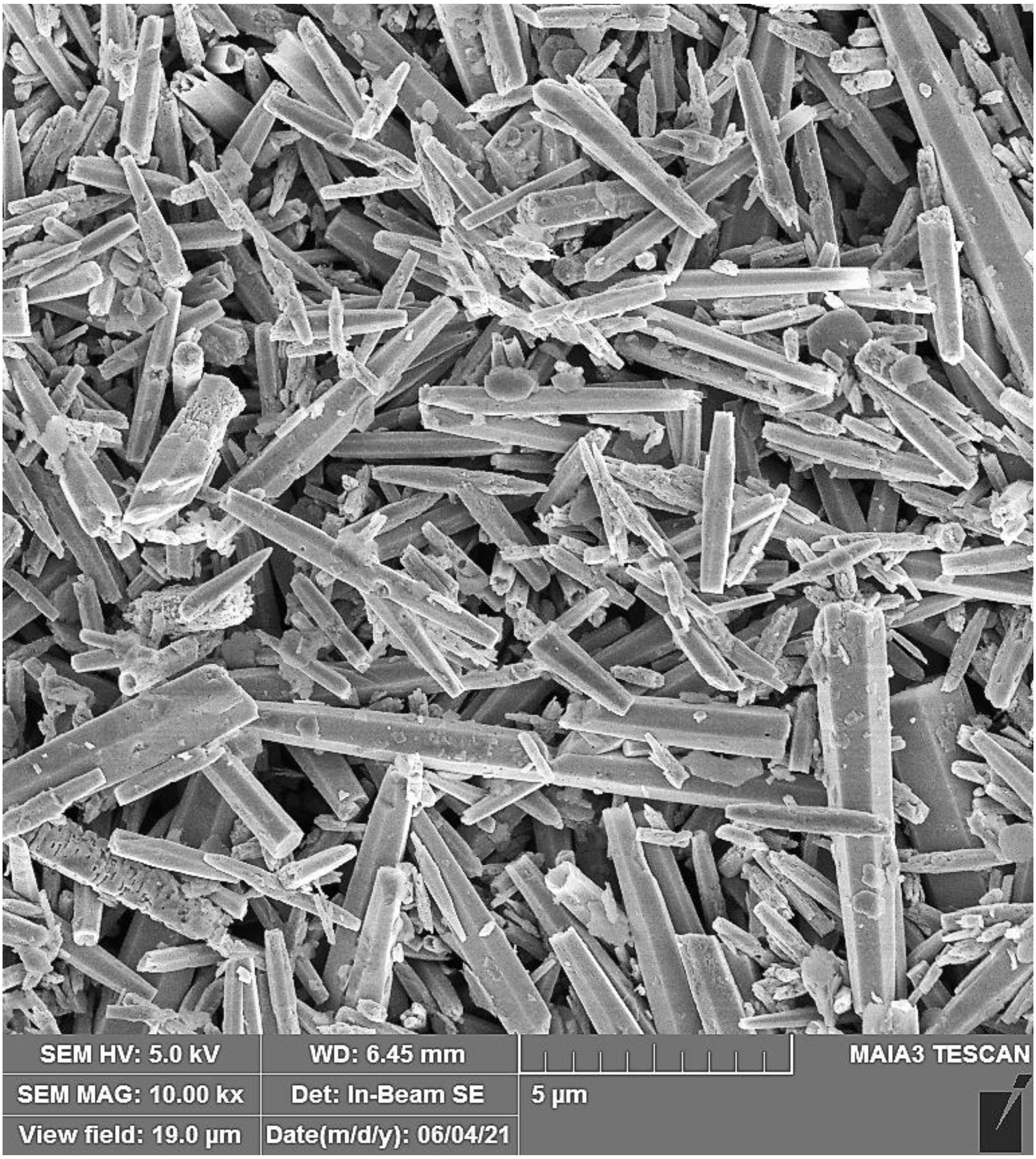


**Figure S4.** Original SEM image of the hydrogen plasma treated ZnO:Mo(0.05%) sample.

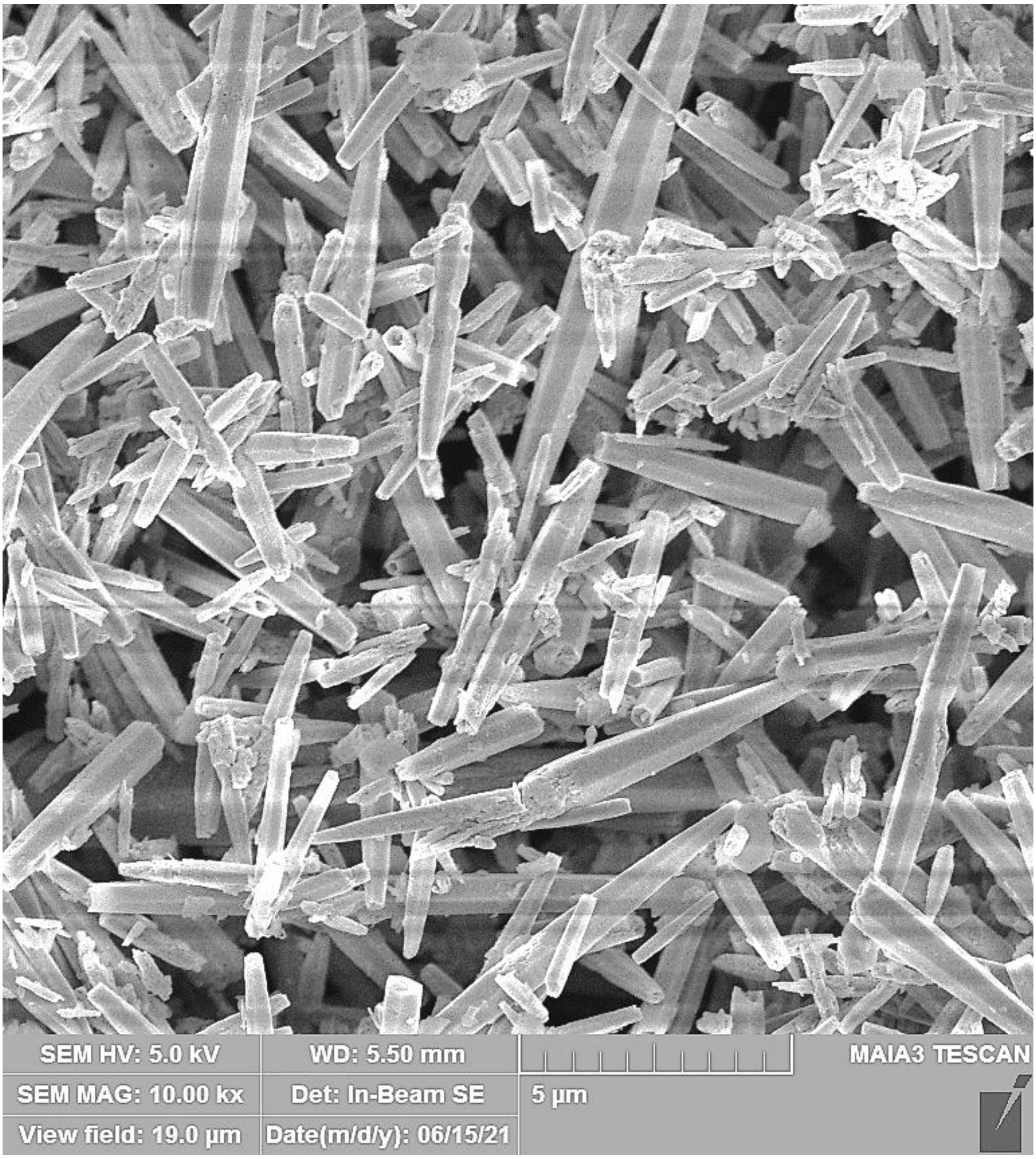


**Figure S5.** Original SEM image of the oxygen plasma treated ZnO:Mo(0.05%) sample.
